# Co-cultivation of *Trichoderma asperellum* GDFS1009 and *Bacillus amyloliquefaciens* 1841 Causes Differential Gene Expression and Improvement in the Wheat Growth and Biocontrol Activity

**DOI:** 10.3389/fmicb.2019.01068

**Published:** 2019-05-16

**Authors:** Valliappan Karuppiah, Jianan Sun, Tingting Li, Murugappan Vallikkannu, Jie Chen

**Affiliations:** ^1^School of Agriculture and Biology, Shanghai Jiao Tong University, Shanghai, China; ^2^State Key Laboratory of Microbial Metabolism, Shanghai Jiao Tong University, Shanghai, China

**Keywords:** co-cultivation, *T. asperellum*, *B. amyloliquefaciens*, plant growth, biocontrol

## Abstract

In an effort to balance the demands of plant growth promoting and biological control agents in a single product, the technology on the co-cultivation of two microbes, *Trichoderma asperellum* GDFS1009 and *Bacillus amyloliquefaciens* 1841 has been developed and demonstrated its effectiveness in synergistic interactions and its impact on the plant growth and biocontrol potential. In this study, optimization of *T. asperellum* and *B. amyloliquefaciens* growth in a single medium was carried out using response surface methodology (RSM). The optimal medium for enhanced growth was estimated as 2% yeast extract, 2% molasses and 2% corn gluten meal. *T. asperellum* evolved the complicated molecular mechanisms in the co-culture by the induction of BLR-1/BLR-2, VELVET, and NADPH oxidases genes. In performance with these genes, conserved signaling pathways, such as heterotrimeric G proteins and mitogen-activated protein kinases (MAPKs) had also involved in this molecular orchestration. The co-cultivation induced the expression of *T. asperellum* genes related to secondary metabolism, mycoparasitism, antioxidants and plant growth. On the other hand, the competition during co-cultivation induced the production of new compounds that are not detected in axenic cultures. In addition, the co-culture significantly enhanced the plant growth and protection against *Fusarium graminearum*. The present study demonstrated the potential of co-cultivation technology could be a used to grow the *T. asperellum* GDFS1009 and *B. amyloliquefaciens* 1841 synergistically to improve the production of mycoparasitism related enzymes, secondary metabolites, and plant growth promoting compounds to significantly enhance the plant growth and protection against plant pathogens.

## Introduction

The genus *Trichoderma* is recognized for its biocontrol function against the fungal phytopathogens (Contreras-Cornejo et al., 2016) such as *Rhizoctonia solani*, *Botrytis cinerea*, *Sclerotium sclerotiorum*, *Pythium* spp., and *Fusarium* spp. *Trichoderma* mycoparasitism involves the detection, attachment and coiling in the region of the host hyphae, and release the antibiotic metabolites and cell-wall degrading enzymes (Zeilinger et al., 2016). The *Trichoderma* species produce several compounds, such as isonitrile, diketopiperazines, sesquiterpenes, polyketides, alkylpyrones, and peptaibols (Patil et al., 2016). However, *Trichoderma asperellum*, a less studied fungus, is also a successful biological control agent against a wide range of plant pathogens (Marcello et al., 2010; Wu et al., 2017). *T. asperellum* has been identified to antagonize the pathogen *Fusarium oxysporum*, *Corynespora cassiicola*, and *Curvularia* *aeria*. Further, its efficacy has also been demonstrated (Baiyee et al., 2019; Veenstra et al., 2019). *B. amyloliquefaciens* is a root-colonizing biocontrol bacterium and is employed to battle several phyto-pathogens. It has been shown advantages to plants through the soil and hydroponic applications (Chowdhury et al., 2015). It inhibits the growth of pathogens, and delay the infection, though reasonable exclusion and competition with the pathogen (Huang et al., 2014; Tan et al., 2016). *Bacillus amyloliquefaciens* have also been found to antagonize *B. cinerea* and *Fusarium solani* (Freitas et al., 2019). Since the *T. asperellum* and *B. amyloliquefaciens* have the various benefits to the plant growth and development, in the present investigation, we aimed to develop the consortium of *T. asperellum* and *B. amyloliquefaciens* through the co-cultivation technology.

Despite, the biocontrol and plant growth promoting mechanism of *B. amyloliquefaciens* and *T. asperellum* are different; the co-cultivation technology could enable both organisms to improve the biocontrol and plant growth. Microbial co-cultivation has been effectively used to induce the expression of several cryptic pathways. Co-cultivation increase the production of new secondary metabolites through the activation of signaling molecules (Wakefield et al., 2017). It also leads to the synthesis of enzymes to increase the metabolite production (Abdelmohsen et al., 2015). Wu et al. (2018) found that the antibiosis of *B. amyloliquefaciens* ACCC11060 and *T. asperellum* GDFS1009 was increased under co-cultivation conditions. Hence, in the present study, the optimal medium for *B. amyloliquefaciens* 1841 and *T. asperellum* GDFS1009 cultivation was developed using response surface methodology. Further, the differential expression of sporulations, enzymes and secondary metabolites related genes were analyzed and tested its efficacy on the plant growth and biocontrol activity.

## Materials and Methods

### Strains

The biocontrol fungus *T. asperellum* GDFS1009 was obtained from the China General Microbiological Culture Collection Center (CGMCC NO. 9512) Beijing, China. The biocontrol bacterium *B. amyloliquefaciens* 1841 was obtained from the Laboratory of Microbial Fermentation, Sichuan University, China. *Fusarium graminearum* (wheat head blight and root rot pathogen) *F. graminearum* (maize stack pathogen), *B. cinerea* and *F. oxysporum* were used as a target pathogen for the examination of mycoparasitism activity.

### Optimization of Co-culture Medium

1 ml of *T. asperellum* GDFS1009 inoculums (10^6^ spores/ml) was pre-cultured at different time intervals (0, 4, and 48 h) in 100 mL of TY (Benoit et al., 2015), NB (Peptone 1%; Beef extract 1%; Sodium chloride 0.5%) and YMC broth (Yeast extract 2%; Molasses 1% and Corn Gluten meal 1%), subsequently 100 μl of the *B. amyloliquefaciens* 1841 inoculums (1.0 OD at 600 nm) was inoculated into the preculture medium and incubated at 180 rpm at 28°C for 2 days. 1 ml of *T. asperellum* GDFS1009 and 100 μL of *B. amyloliquefaciens* 1841 were singly inoculated into 100 ml of TY, NB and YMC broth, incubated in shaker for 4 days. After incubation, the broth cultures were serially diluted and plated in the PDA containing streptomycin and chloramphenicol to estimate the growth of fungus. Whereas, the bacterial growth was estimated using the nutrient agar containing nystatin and cycloheximide.

### Experimental Design and Data Analysis

The ingredients of YMC medium on the growth of *T. asperellum* GDFS1009 and *B. amyloliquefaciens* 1841 were enhanced by central composite design (CCD) at five distinct levels. The impact of the yeast extract (X1), molasses (X2) and corn gluten meal (X3) on the growth of *T. asperellum* GDFS1009 and *B. amyloliquefaciens* 1841 (Y) were accessed. The growth of *T. asperellum* GDFS1009 and *B. amyloliquefaciens* 1841 were taken as the average of the duplicate. The experimental designs with the observed and predicted values for growth of *T. asperellum* GDFS1009 and *B. amyloliquefaciens* 1841 were displayed in Table 1. The second order polynomial coefficients were determined and examined using the Minitab package version 18. The general type of the second-degree polynomial equation was resolved as depicted by Karuppiah et al. (2013). Statistical investigation was carried out using Minitab package version 18. The anticipated values for the growth of *T. asperellum* GDFS1009 and *B. amyloliquefaciens* 1841 were confirmed by a trial using the optimum values of the factors obtained from the CCD response optimization.

**Table 1:** Central composite design and results along with the observed and predicted values of the *T. asperellum* and *B. amyloliquefaciens* growth in the co-culture conditions.

Run order	Yeast extract (gm/100 ml)	Molasses (gm/100 ml)	Corn gluten meal (gm/100 ml)	*Trichoderma* growth (CFU × 10^9^)		*Bacillus* growth (CFU × 10^11^)	
				Observed	Predicted	Observed	Predicted
1	1	1	1	5.2	5.184091	14	13.98636
2	3	1	1	5.6	5.609091	14.4	14.41136
3	1	3	1	5.6	5.609091	14.4	14.41136
4	3	3	1	5.5	5.534091	14.3	14.33636
5	1	1	3	5.7	5.684091	14.5	14.48636
6	3	1	3	5.4	5.409091	14.2	14.21136
7	1	3	3	5.6	5.609091	14.4	14.41136
8	3	3	3	4.8	4.834091	13.6	13.63636
9	0	2	2	3.6	3.615909	12.4	12.41364
10	4	2	2	3.3	3.265909	12.1	12.06364
11	2	0	2	5.2	5.215909	14	14.01364
12	2	4	2	5.1	5.065909	13.9	13.86364
13	2	2	0	5	4.990909	13.8	13.78864
14	2	2	4	4.8	4.790909	13.6	13.58864
15	2	2	2	8.2	8.263636	17	17.07955
16	2	2	2	8.3	8.263636	17.1	17.07955
17	2	2	2	8.3	8.263636	17.1	17.07955
18	2	2	2	8.2	8.263636	17	17.07955
19	2	2	2	8.4	8.263636	17	17.07955
20	2	2	2	8.2	8.263636	17.3	17.07955

### Gene Expression Study

The expression of genes related to the sporulation and secondary metabolites is induced by a several environmental stimuli (Netzker et al., 2015). Hence, the expression of the genes related to sporulation (*VEL* 1, *TMK*, *GPR* 1, *BLR* 1, *BLR* 2, and *ENV1*) (Mukherjee et al., 2003; Casas-Flores et al., 2004; Brunner et al., 2008; Bazafkan et al., 2017), mycoparasitism related enzymes (*NAG* 1, *NAG* 2, *PAP* A, *PAP* B, *ECH*, *AF*, *ACC*, *BGN13*, *BGN* 16, and *EG1*) (Chet et al., 2004; Viterbo et al., 2010; Yang, 2017) secondary metabolism (genes encoding three NRPSs, two PKSs, *O*-methyl transferase B, and cytochrome P450) (Mukherjee and Kenerley, 2010) and antioxidants (*NOX* and *CAT*) (Montero-Barrientos et al., 2011) of *Trichoderma.* In addition, macrolactin and difficidin genes (Goodson et al., 2017) of *B. amyloliquefaciens* were studied in the axenic and co-culture of the *T. asperellum* and *B. amyloliquefaciens* grown in the YMC medium at 4th day.

For RNA isolation, the cultures were snap-frozen in liquid nitrogen, ground, and extracted using TRIeasy total RNA extraction reagent (YEASEN) according to the manufacturer’s protocol. For RT-PCR, cDNA was synthesized from 1 μg of RNA using Prime Script RT reagent kit with DNA eraser (Takara). Real-time PCR was performed using Super Real premix plus SYBR green kit (Tiangen Biotech) in the Roche light cycler 96. The PCR conditions were as follows: 95°C for 5 min, followed by 40 cycles of 95°C for 10 s, 60°C for 30 s, and 72°C for 30 s. Each PCR reaction was carried out in triplicate. Subsequently, threshold-dependent cycling, melting was executed from 60 to 95°C at 0.2°C/s melt rate with a smooth curve. Primer specificity was confirmed through melt curve study. *C*q values were normalized to the 18S rRNA and 16S rRNA *C*q values for 2^−ΔΔCT^ relative quantification. 18S rRNA and 16S rRNA genes were used as the reference gene for *Trichoderma* and *Bacillus*, respectively. The monocultures were treated as the control. The sequences of the primers used for real time PCR are given the Supplementary Table S1.

#### Enzyme Assay

The axenic and co-culture of *T. asperellum* and *B. amyloliquefaciens* were cultured in YMC medium. The cultures were vacuum-filtrated, and the filtrate was used as the crude protein extract. Each enzyme activity was assayed twice with three replicates. Chitinase activity was assessed using a chitinase enzyme Activity Determination Kit (Shanghai Cablebridge Biotechnology Co., Ltd.), according to the manufacturer’s instructions. An enzymatic unit was defined as that releasing 1.0 mmol of *p*-nitrophenol from the appropriate substrate per min at 37 °C. Activities of β-1,3-glucanase and cellulase were measured using laminarin and carboxy methyl cellulose as the substrates, respectively. β-1,3-glucanase and cellulase activity was assessed using a β-1,3-glucanase and Cellulase enzyme Activity Determination Kit (Shanghai Cablebridge Biotechnology Co., Ltd.), respectively, according to the manufacturers instruction. The protease activity was assayed using casein as a substrate using the Neutral Protease Activity Determination Kit (Shanghai Cablebridge Biotechnology Co., Ltd.) according to the manufacturers instruction.

### Analysis Secondary Metabolites Using GC MS Analysis

The axenic and co-culture of *T. asperellum* GDFS1009 and *B. amyloliquefaciens* 1841 were grown in shake flask culture on YMC medium (Yeast extract 2%; Molasses 2% and Corn Gluten meal 2%). After fermentation for 7 days at 28°C at 180 rpm/min, fermentation broths were filtered. To extract the secondary metabolites from the axenic and co-culture of *T. asperellum* GDFS1009 and *B. amyloliquefaciens* 1841 fermented broth, the filtered liquid culture (4 L) was mixed with equal volume of ethyl acetate (4 L) and shaken vigorously for 5 min in separating flask. After resolving for 1 h, the organic phase was separated and concentrated to dryness by means of rotary evaporator.

Bioactive metabolites present in the ethyl acetate extract of the axenic and co-culture was resolved using gas chromatography*–* mass spectroscopy systems. The 7890A gas chromatograph and a 5975C mass spectrometer (Agilent Technologies, Milan, Italy) were utilized to perform GC-MS investigation. The HP-5 GC column (30 m × 0.32 mm i.d., 0.50 μm film thickness) were utilized to distinguish the secondary metabolites as described by Srinivasa et al. (2017). The helium gas at the stream rate of 1 ml/minute was utilized to distinguish the secondary metabolites. The recognized metabolites were compared with the mass spectra and the information system libraries of Wiley-2009 and NIST-2007.

### Antagonism Assays

Fifty ml of the axenic and co-culture fermented broth were filtered and blended into 100 ml of PDA medium and poured into the petri dishes. The pathogens [*F. oxysporum*, *Fusarium graminearum* (wheat head blight and root rot pathogen and maize stack pathogen), and *B. cinerea*] were inoculated into the petri dishes and incubated at 28°C for 5 days.

The abilities of the axenic and co-culture respond to the inhibition of pathogen growth were studied using the simulated antagonistic assay (Mendoza-Mendoza et al., 2003). Briefly, the axenic and co-cultures were prepared in YMC medium, washed and transferred into 4 days old *F. graminearum* (wheat head blight and root rot pathogen) culture grown in the Vogel’s minimal medium (VMS) medium (Davis, 2000). After 24 h incubation with shaking, the spores of *F. graminearum*, *T. asperellum* GDFS1009 and *B. amyloliquefaciens* 1841 were counted using the light microscope. The axenic and co-cultures were harvested, frozen, and the RNA was extracted. Induction of the mycoparasitism-related gene prb1/sp1 encoding serine protease, which response to *F. graminearum* (Pozo et al., 2004) was assessed by quantitative RT-PCR.

### Seed Germination Assay

The spore suspension was adjusted to 5 × 10^9^ ml and supplemented with 0.15% (v/v) of seed coating agents (Jilin Bada Pesticide Co. Ltd.) as an adhesive. The 20 g of wheat seeds (*Ningmai* 13) were disinfected and treated with the 2 ml of each axenic and co-culture spore suspension. Ten seeds from every treatment were set in to the Petri dish containing Whatman filter paper in triplicates. Sterilized distilled water was added to maintain the moisture and the seeds were sprouted in dark at 25°C. The developmental parameters, for example, shoot length, root length, seedling length, seedling wet weight, seedling dry weight and vigor index were recorded after 5 days.

### Biocontrol Assays Under Greenhouse Conditions

The wheat seeds treated with axenic and co-culture were germinated and planted in pot soil, and grown at 28°C for 12 h under lights, and at 24°C in the dark for 12 h. One month seedlings were prepared for the assay. The spore suspension of axenic and co-culture grew in YMC medium was collected and diluted to a concentration of 1 × 10^6^ conidia ml^−1^ with sterile distilled water and evenly applied to the potting soil. After treatment with axenic and co-cultures for 7 days, the conidial suspension of *F. graminearum* was prepared to a final concentration of 1 × 10^6^ conidia ml^−1^ and applied to the potting soil. Inoculation with the only pathogen and distilled water was taken as controls. The plant growth promoting and biocontrol parameters such as shoot length, root length, wet weight, dry weight and number of grains and disease index was calculated as described by Wang et al. (2015).

### Statistical Analysis

All these experiments were carried out using three replicates and were repeated at least twice, with reproducible results. The figures were plotted using microsoft office excel and origin 6.0 with standard error bars. The medium was optimized statistically using Minitab 18 software. Values were given as mean ± SEM. For multiple comparisons, two-way ANOVA with *post hoc* LSD, Duncan and Bonferroni were performed using the SPSS 2.0. Student’s *t*-test was used to analyze the gene expression between the axenic and co-cultures using the SPSS 2.0. *P* < 0.05 was considered as significant.

## Results

### Screening of Different Media for the Growth of *T. asperellum* (TA) and *B. amyloliquefaciens* (BA) in the Axenic and Co-culture

The growth of *T. asperellum* (TA) and *B. amyloliquefaciens* (BA) in the axenic and co-culture conditions in complete and chemically defined culture media were shown in Table 2. The maximum growth of *T. asperellum* and *B. amyloliquefaciens* was observed in the 48th hour TA pre-culture inoculated with the BA in YMC medium followed by NB and TY medium. Whereas, the growth of *Trichoderma* was affected in the co-culture of *B. amyloliquefaciens* inoculated into the 0th hour TA pre-culture. The growth of *T. asperellum* and *B. amyloliquefaciens* axenic cultures were increased in the YMC medium. The results indicated that axenic and co-culture of *T. asperellum* and *B. amyloliquefaciens* were enhanced in the YMC medium. Further, the sequential inoculation of *T. asperellum* and *B. amyloliquefaciens* was effective than that of co- inoculation.

**Table 2:** Growth rate of *T. asperellum* and *B. amyloliquefaciens* in axenic and co-culture on different media.

Experiments	*Trichoderma* (CFU)	*Bacillus* (CFU)
**YMC**		
*Bacillus amyloliquefaciens* (BA)		4.8 × 10^12^ ± 0.6
*T. asperellum* (TA)	1 × 10^8^ ± 0.02	^−^
*0th hour* (TA) pre-culture + (BA)	0	2.27 × 10^11^ ± 0.3
*24th hour* (TA) pre-culture + (BA)	1 × 10^8^ ± 0.1	6 × 10^7^ ± 0.09
*48th hour* (TA) pre-culture + (BA)	7 × 10^9^ ± 0.3	7 × 10^11^ ± 0.08
**NB**		
*Bacillus amyloliquefaciens* (BA)		7.1 × 10^10^ ± 0.03
*T. asperellum* (TA)	3 × 10^6^ ± 0.4	^−^
*0th hour* (TA) pre-culture + (BA)	0	14 × 10^7^ ± 0.2
*24th hour* (TA) pre-culture + (BA)	10 × 10^5^ ± 0.3	8 × 10^8^ ± 0.5
*48th hour* (TA) pre-culture + (BA)	12 × 10^6^± 0.07	8 × 10^9^ ± 0.6
***TY***		
*Bacillus amyloliquefaciens* (BA)		14 × 10^10^ ± 0.4
*T. asperellum* (TA)	4 × 10^7^ ± 0.07	^−^
*0th hour* (TA) pre-culture + (BA)	0	3.3 × 10^10^ ± 0.04
*24th hour* (TA) pre-culture + (BA)	8 × 10^6^± 0.1	1 × 10^8^ ± 0.08
*48th hour* (TA) pre-culture + (BA)	1 × 10^7^ ± 0.2	5 × 10^9^ ± 0.3

*Results are means of five replicates for each experiments; the value given is the standard error of the mean.*

### Optimization of Medium Components Using Central Composite Design

Preliminary experiments showed that YMC medium influenced the growth of *T. asperellum* and *B. amyloliquefaciens* in both axenic and co-culture conditions. So, Central Composite Design was used to optimize the medium components of YMC medium. The consequence of yeast extract, molasses and corn gluten meal at five levels and their relations on the growth of *T. asperellum* and *B. amyloliquefaciens* was shown in Table 1.

Analysis of variance (ANOVA) for the growth of *T. asperellum* and *B. amyloliquefaciens* was depicted in Table 3. The results revealed that *R*^2^ was 0.9992 and 0.9985, demonstrating that the experiment was fitted and elucidate 99.92 and 99.85% of the variability on the growth of *T. asperellum* and *B. amyloliquefaciens*, respectively, in co-culture conditions. Similarly, *F*-Test of the regression was also reported to be significant. Whereas, the lack of fit, 0.956 and 0.988 obtained for *T. asperellum* and *B. amyloliquefaciens* were not significant. These outcomes demonstrate that the model picked clarified the impacts of yeast extract, molasses and corn gluten meal on the growth of *T. asperellum* and *B. amyloliquefaciens* in the co-cultivation. The second-order polynomial equation on the growth of *T. asperellum* (Y1) and *B. amyloliquefaciens* (Y2) were as follows.

**Table 3:** Statistical analysis of central composite design showing coefficient values, *t*- and *p*-values for each variable on growth of *T. asperellum* and *B. amyloliquefaciens.*

Source	DF	Adj SS	Adj MS	*F*-value	*P*-value
Model	9^A^	51.0205^A^	5.6689^A^	1433.52^A^	0^A^
	9^B^	51.4743^B^	5.7194^B^	760.28^B^	0^B^
Linear	3^A^	0.185^A^	0.0617^A^	15.59^A^	0^A^
	3^B^	0.185^B^	0.0617^B^	8.2^B^	0.005^B^
Yeast extract	1^A^	0.1225^A^	0.1225^A^	30.98^A^	0^A^
	1^B^	0.1225^B^	0.1225^B^	16.28^B^	0.002^B^
Molasses	1^A^	0.0225^A^	0.0225^A^	5.69^A^	0.038^A^
	1^B^	0.0225^B^	0.0225^B^	2.99^B^	0.114^B^
Corn gluten meal	1^A^	0.04^A^	0.04^A^	10.11^A^	0.01^A^
	1^B^	0.04^B^	0.04^B^	5.32^B^	0.044^B^
Square	3^A^	50.3405^A^	16.7802^A^	4243.26^A^	0^A^
	3^B^	50.7943^B^	16.9314^B^	2250.7^B^	0^B^
Yeast extract ∗ Yeast extract	1^A^	36.5494^A^	36.5494^A^	9242.37^A^	0^A^
	1^B^	36.8255^B^	36.8255^B^	4895.23^B^	0^B^
Molasses ∗ Molasses	1^A^	15.3237^A^	15.3237^A^	3874.95^A^	0^A^
	1^B^	15.5026^B^	15.5026^B^	2060.77^B^	0^B^
Corn gluten meal ∗ Corn gluten meal	1^A^	17.8755^A^	17.8755^A^	4520.23^A^	0^A^
	1^B^	18.0687^B^	18.0687^B^	2401.88^B^	0^B^
2-Way Interaction	3^A^	0.495^A^	0.165^A^	41.72^A^	0^A^
	3^B^	0.495^B^	0.165^B^	21.93^B^	0^B^
Yeast extract ∗ Molasses	1^A^	0.125^A^	0.125^A^	31.61^A^	0^A^
	1^B^	0.125^B^	0.125^B^	16.62^B^	0.002^B^
Yeast extract ∗ Corn gluten meal	1^A^	0.245^A^	0.245^A^	61.95^A^	0^A^
	1^B^	0.245^B^	0.245^B^	32.57^B^	0^B^
Molasses ∗ Corn gluten meal	1^A^	0.125^A^	0.125^A^	31.61^A^	0^A^
	1^B^	0.125^B^	0.125^B^	16.62^B^	0.002^B^
Error	10^A^	0.0395^A^	0.004^A^		
	10^B^	0.0752^B^	0.0075^B^		
Lack-of-fit	5^A^	0.0062^A^	0.0012^A^	0.19^A^	0.956^A^
	5^B^	0.0069^B^	0.0014^B^	0.1^B^	0.988^B^
Pure error	5^A^	0.0333^A^	0.0067^A^		
	5^B^	0.0683^B^	0.0137^B^		
Total	19^A^	51.06^A^			
	19^B^	51.5495^B^			

*^A^Growth rate of T. asperellum, ^B^growth rate of B. amyloliquefaciens.*

(1) $$\begin{array}{c} \text{Y}1=-4.405+5.3352\text{ X}1+3.5852\text{ X}2+3.9227\text{ X}3\\ \;-\;1.2057\text{ X}1*\text{X}1-0.7807\text{ X}2*\text{X}2\\ \;-\;0.8432\text{ X}3*\text{X}3-0.1250\text{ X}1*\text{X}2\\ \;-\;0.1750\text{ X}1*\text{X}3-0.1250\text{ X}2*\text{X}3 \end{array}$$

(2) $$\begin{array}{c} \text{Y}2=4.357+5.353\text{ X}1+3.603\text{ X}2+3.941\text{ X}3\\ \;-\;1.2102\text{ X}1*\text{X}1-0.7852\text{ X}2*\text{X}2\\ \;-\;0.8477\text{ X}3*\text{X}3-0.1250\text{ X}1*\text{X}2\\ \;-\;0.1750\text{ X}1*\text{X}3-0.1250\text{ X}2*\text{X}3 \end{array}$$

Where Y1 and Y2 is the growth of *T. asperellum*, and *B. amyloliquefaciens*, respectively; X1, X2, and X3 are the coded values of the test variables yeast extract, molasses, and corn gluten meal, respectively.

**FIGURE 1:**
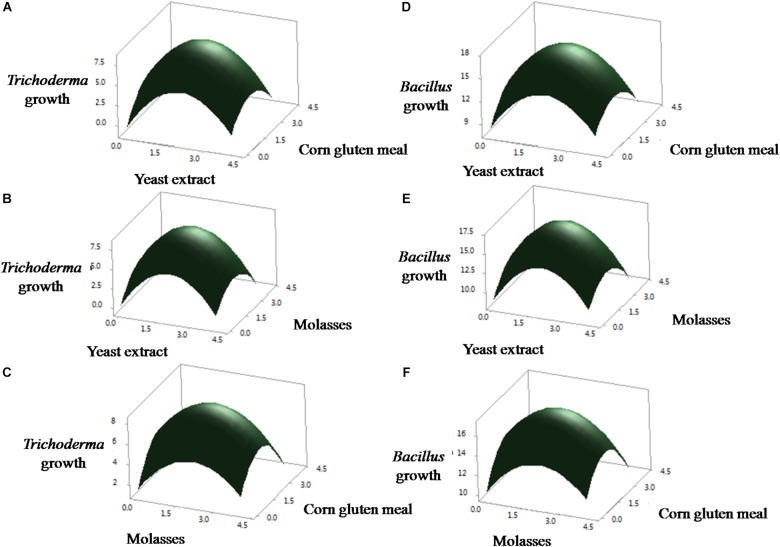
Statistical optimization of the *T. asperellum* and *B. amyloliquefaciens* growth in co-culture using RSM. **(A–C)** Effect of yeast extract, corn gluten meal and molasses on the growth of *T. asperellum*. **(D–F)** Effect of yeast extract, corn gluten meal and molasses on the growth of *B. amyloliquefaciens*.

The results demonstrated that yeast extract, molasses, and corn gluten meal had positive effect on the growth of *T. asperellum*, and *B. amyloliquefaciens*. Among the three factors tested, yeast extract had the most noteworthy effect on the growth of *T. asperellum*, and *B. amyloliquefaciens*, as given by the direct coefficient. Followed by, molasses and corn gluten meal showed the positive effect on the growth of *T. asperellum*, and *B. amyloliquefaciens*. These variables likewise demonstrated huge negative quadratic effects on the growth of *T. asperellum*, and *B. amyloliquefaciens*, demonstrating that growth of *T. asperellum*, and *B. amyloliquefaciens*, increased and decreased based on the ideal level of each factors. The association between these parameters was likewise noteworthy, as appeared low *P*-values (*P* < 0.05) for intelligent terms. Fitted reaction for the above deterioration was plotted (Figure 1). The charts were plotted for 2 factors while keeping the other on central level. The surface plots confirmed that factors are unimodal in nature. The optimal concentrations of yeast extract, molasses, and corn gluten meal were anticipated in genuine units and they were 2.0 g each with a growth of *T. asperellum*, and *B. amyloliquefaciens*. The confirmation experiments showed that colony count of *T. asperellum* and *B. amyloliquefaciens* were 8.7 × 10^9^ and 17.3 × 10^11^, respectively. This was higher than the anticipated results and confirmed the accuracy of the model.

### Differential Gene Expression in the Co-culture of *T. asperellum* and *B. amyloliquefaciens*

Distinct molecular expressions were observed for each species under co-cultivation conditions. This was measured by means of qRT-PCR from co-cultivation treatments contrasted to their particular axenic controls. Differentially expressed genes were characterized as fold change among axenic and co-cultivation. The number of differentially expressed genes belonging to each functional category was normalized against the reference genes, 18S rRNA and 16srRNA genes for *T. asperellum* and *B. amyloliquefaciens*, respectively.

**FIGURE 2:**
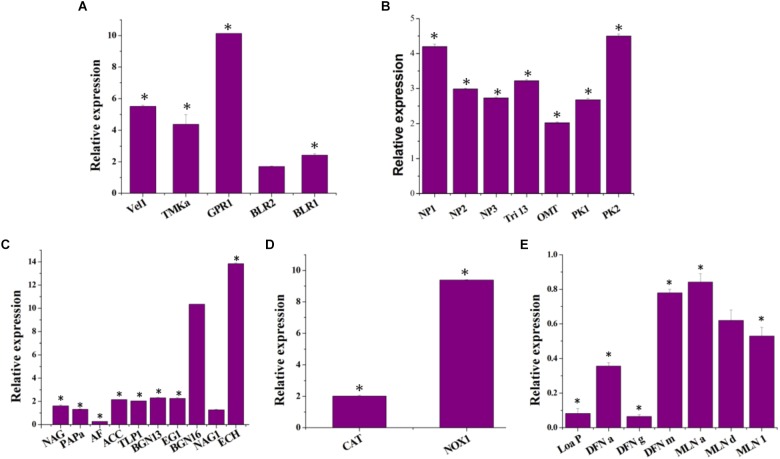
Impact of co-cultivation on the transcript levels of **(A)** the *T. asperellum* morphology related genes [Mitogen-Activated Protein Kinase (*TMKa*), G protein receptor 1 (GPR1), blue-light-regulated genes (BLR1 and BLR2) and ENVOY (ENV 1)], **(B)** secondary metabolism related genes [non-ribosomal peptide synthetase (NP1 and NP2), Putative ferrichrome synthetase (NP3), Cytochrome P450 (Tri 13) 1, *O*-methyl transferase (OMT) and Polyketide synthetase (PK1 and PK2)]; **(C)** mycoparasitism-related genes [chitinase (ech42), β-1,3-glucanase (bgn13.1), β1,6-glucanase (bgn16.1), β-1,4-glucanase (egl), *N*-acetyl-glucosaminidases (nag1 and nag2), aspartyl protease (Pap A), trypsin-like protease (TLP 1) and α–L–arabinofuranosidases (AF)]; and plant growth promoting enzyme [1–Aminocyclopropane–1–carboxylate (ACC) deaminase (ACC)]. **(D)** Anti-oxidant genes [NADPH oxidase (NOX), catalase (CAT)] and **(E)** genes encoding *B. amyloliquefaciens* macrolactin and difficidin in the co-culture [intrinsic terminators located within the polyketide synthase (PKS) gene cluster encoding for the antibiotic difficidin (*dfn*) (*Loa P*), beginning, middle and end of the difficidin operon (dfnA, dfnG, and dfnM); beginning, middle and end of the macrolactin operon (MLN a, MLN d, and MLN i)]. Data resulted from biological triplicate cultures with qPCR technical duplicates. The value in parentheses is the standard error of the mean. ^∗^represent significant differences between the axenic and co-culture (*P* < 0.05).

Differences in gene functions were observed between axenic and co-culture. Co-cultivation resulted in the upregulation of *T. asperellum* genes involved in the sporulation, secondary metabolism, antagonism and plant growth promoting related enzymes and antioxidants. The sporulation-related genes such as Velvet (*Vel* 1), Mitogen-Activated Protein Kinase (*TMKa*), G protein receptor 1 (*GPR1*), blue-light-regulated genes (*BLR1* and *BLR2*) were upregulated in *T. asperellum* in response to *B. amyloliquefaciens* (Figure 2A). Similarly, *T. asperellum* genes involved in secondary metabolisms such as non-ribosomal peptide synthetase (*NP1* and *NP2*), Putative ferrichrome synthetase (*NP3*), Cytochrome P450 (*Tri* 13) 1, *O*-methyl transferase (*OMT*) and Polyketide synthetase (*PK1* and *PK2*) were also upregulated (Figure 2B).

To determine whether co-cultivation is involved in the regulation of genes encoding the pathogen cell wall degrading enzymes, the transcription of six mycoparasitism-related genes *Ech42* (chitinase), *BGN13*.1 (β-1,3-glucanase), *BGN16*.1 (β1,6-glucanase), *Egl* (β-1,4-glucanase) and *NAG1* and *NAG2* (*N*-acetyl-glucosaminidases) were measured in axenic and co-culture. The expression of *Ech42* has upregulated 13.83-fold in co-culture compared to the axenic culture of *T. asperellum* (Figure 2C). *BGN13* and *BGN16* gene expression levels were also induced by *B. amyloliquefaciens* (Figure 2C). In Co-culture *NAG1*, *NAG2*, and *EG*1 transcription were increased 1.26, 1.6, and 2.25 folds compared to the axenic culture. The expression of aspartyl protease (*PAP* A), trypsin-like protease (*TLP* 1) and 1-aminocyclopropane-1-carboxylate deaminase (*ACC*) were up-regulated 1.31-, 2.02-, and 2.14-folds, respectively. As shown in Figure 2D, the highest transcript levels of NADPH oxidases (*NOX*) and catalase (*CAT*) were detected in the co-cultivation. *B. amyloliquefaciens* showed transcriptional responses to competitive co-cultivation (Figure 2E). 2^−ΔΔCT^ of the differentially expressed macrolactin and difficidin genes of *B. amyloliquefaciens* were less than 1 (Figure 2E), which reveals that these genes were down-regulated in response to competitive co-cultivation.

### Induction of Enzyme Production by the Co-cultivation of *T. asperellum* and *B. amyloliquefaciens*

**FIGURE 3:**
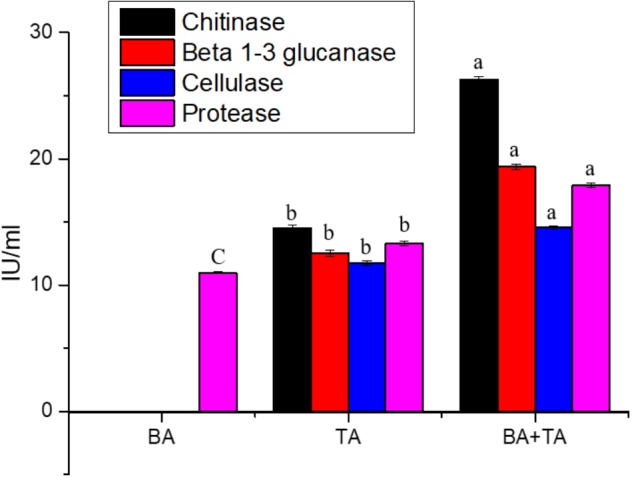
Enzyme activities associated with culture filtrates from the axenic and co-culture of *T. asperellum* and *B. amyloliquefaciens* grown on YMC medium. Results are means of five replicates for each treatment; the value in parentheses is the standard error of the mean. Different letters above the bars are significantly different (*P* < 0.05) based on the ANOVA.

Taking into account on the enhanced gene expression of enzymes related to mycoparasitism in co-culture, we compared the chitinase, β 1–3 glucanase, cellulase and protease activities in the axenic and co-culture medium. As shown in Figure 3, higher levels of chitinase activity were detected in the co-culture than in the axenic culture of *T. asperellum*; these differences were coinciding with the gene expression study. Whereas, chitinase, β 1–3 glucanase and cellulase activities were not detected in the axenic culture of *B. amyloliquefaciens*.

### Induction of Secondary Metabolites by the Co-cultivation of *T. asperellum* and *B. amyloliquefaciens*

**FIGURE 4:**
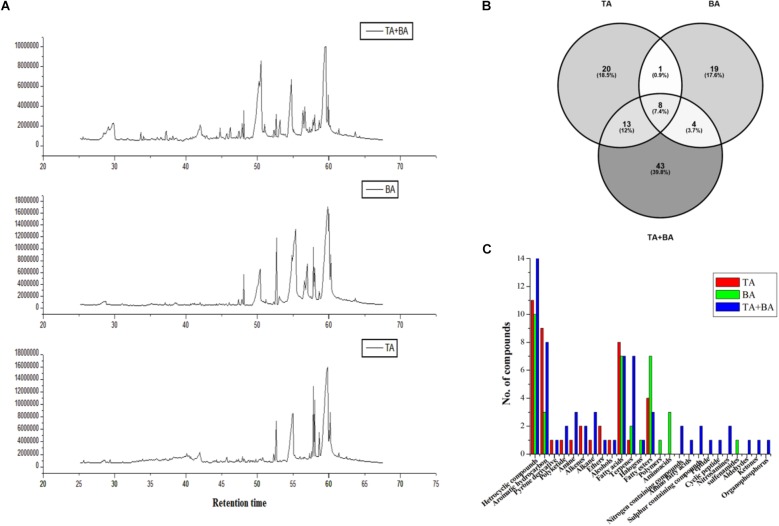
Metabolites differences between the axenic and co-culture of *T. asperellum* and *B. amyloliquefaciens.* **(A)** Differences in the GC MS spectrum **(B)** total number of metabolites detected in the axenic and co-culture **(C)** metabolites class differences in the axenic and co-culture.

We hypothesized that axenic and co-culture differs from one another in their production of secondary metabolites (Figure 4A). After fermentation in YMC medium, the secondary metabolites were extracted using ethyl acetate and analyzed via gas chromatography-mass spectrometry. The compounds with various classes such as Heterocyclic compounds, Aromatic hydrocarbon, Pyrone derivative, Polyketide, Amine, Alkenes, Alkane, Ethers, Alcohols, Fatty acids, Terpenes, Halogens, Fatty esters, Polymers, Amino acids, Nitrogen-containing compounds, Amino fatty acids, Sulfur-containing compounds, Peptide, Cyclic peptide, Nitrosamines, sulfenamides, Aldehydes, Ketones, and Organophosphorus were obtained (Supplementary Table S2). There was notable variability in the number of metabolites between axenic and co-culture. We observed 68 metabolites in the co-culture, which was higher than the axenic culture of *T. asperellum* (42) and *B. amyloliquefaciens* (32) (Figure 4B,C).

### Induction of Antagonistic Activity by the Co-cultivation of *T. asperellum* and *B. amyloliquefaciens*

**FIGURE 5:**
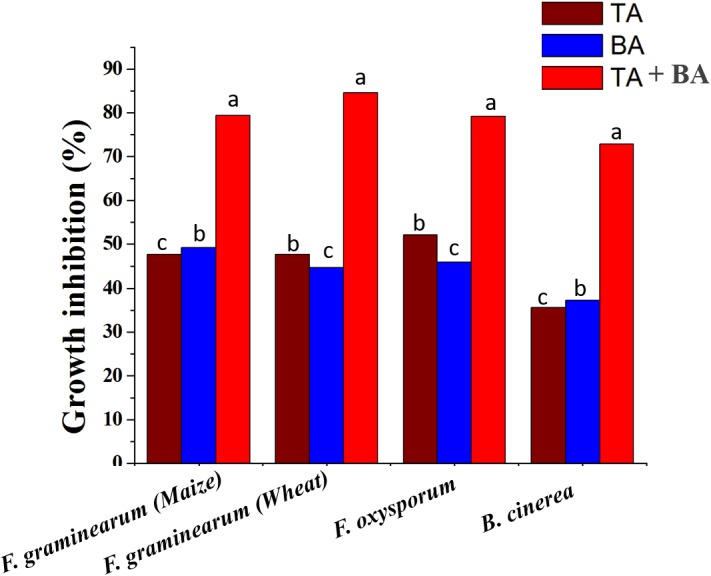
The inhibitory spectrum of axenic and co-culture of *T. asperellum* and *B. amyloliquefaciens* fermentation liquor against plant pathogenic fungi. Results are means of five replicates for each treatment; the value in parentheses is the standard error of the mean. Different letters above the bars are significantly different (*P* < 0.05) based on the ANOVA.

To determine whether the product of axenic and co-culture is involved in the antagonistic capacity against phytopathogenic fungi, PDA or PDA supplemented axenic and co-culture filtrate plates were inoculated in the center with mycelial plugs of *F. graminearum* (wheat head blight and root rot pathogen and maize stack pathogen), *F. oxysporum* and *B. cinerea*. The radial growth of *F. graminearum* was highly inhibited by both co-culture filtrates followed by the axenic culture filtrate of *T. asperellum*, and *B. amyloliquefaciens*, compared to its respective control. Similarly, the radial growth of *F. oxysporum* and *B. cinerea* was highly inhibited by the co-culture filtrates (Figure 5 and Supplementary Figure S1).

**FIGURE 6:**
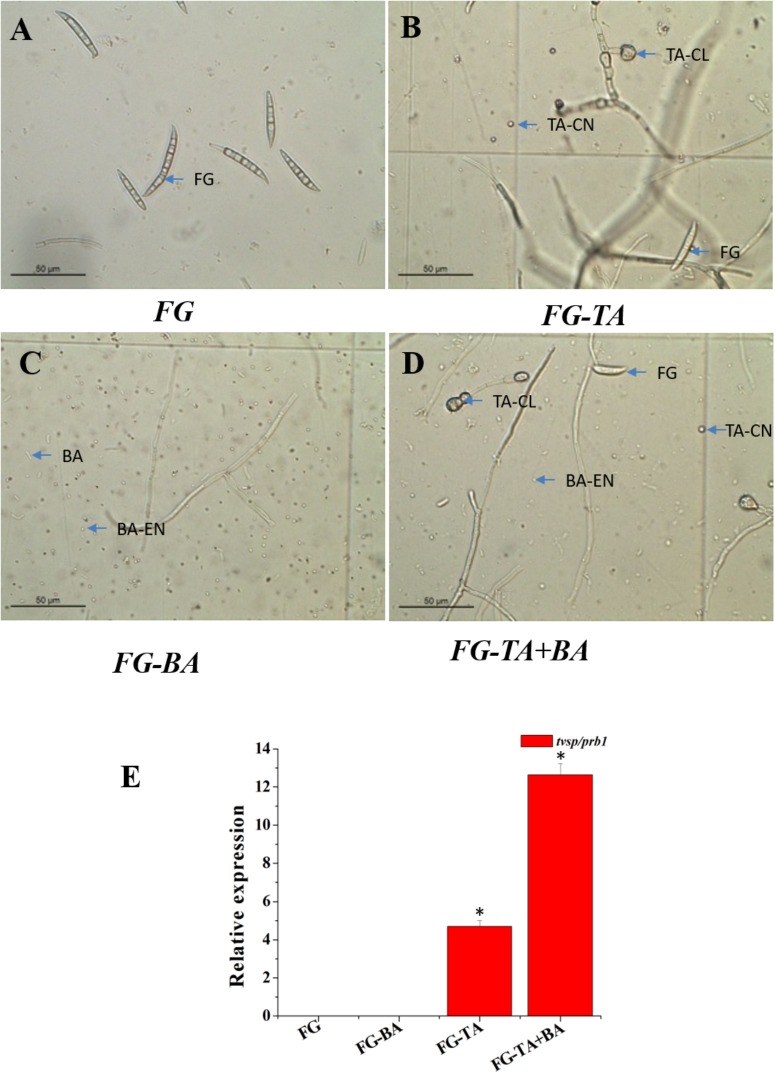
Antagonist interactions between the axenic and co-culture in stimulated antagonism assay against *F. graminearum* (wheat head blight and root rot pathogen). **(A)** Spores of *F. graminearum* (FG). **(B)** Sporulation of *F. graminearum* (FG) in response to the axenic culture of *T. asperellum* (TA). **(C)** Sporulation of *F. graminearum* (FG) in response to the axenic culture of *B. amyloliquefaciens* (BA). **(D)** Sporulation of *F. graminearum* (FG) in response to the Co- culture of *T. asperellum* (TA) and *B. amyloliquefaciens* (BA). **(E)** Induction of serine protease (*tvsp1/prb1*) expression in a stimulated antagonism assay (RT-PCR). *F. graminearum* spores (FG), *T. asperellum* conidiospores (TA-CN), *T. asperellum* chlamydospores (TA-CL), *B. amyloliquefaciens* (BA), *B. amyloliquefaciens* endospores (BA-EN). ^∗^represent significant differences between the axenic and co-culture (*P* < 0.05).

In the stimulated antagonism assay, the spore count of *F. graminearum* (wheat head blight and root rot pathogen) was reduced to 7^∗^10^3^ and 4.5^∗^10^2^ in the TA and TA + BA, respectively (Table 4). The inhibition of the *F. graminearum* was increased in the co-culture conditions (Figure 6A–D). The growth inhibition of *F. graminearum* by the axenic and co-culture of *T. asperellum* and *B. amyloliquefaciens* were correlated with the expression of mycoparasitism related gene *tvsp1/prb1* (coding a serine protease). The relative gene expression of *tvsp1/prb1* gene was highly induced in the co-culture of TA + BA compared to the TA (Figure 6E).

**Table 4:** Antagonist interactions between the axenic and co-culture in stimulated antagonism assay against *F. graminearum* (wheat head blight and root rot pathogen).

Experiments	*T. asperellum* (spores/ml)		*F. graminearum (spores/ml)*	*B. amyloliquefaciens (CFU/ml)*
	Chlamydospores	Conidiospores		
FG	–	–	1.5 × 10^7^ ± 0.6	–
FG + BA	–	–	0	5.7 × 10^12^ ± 0.4
FG + TA	4.5 × 10^7^ ± 0.5	1.2 × 10^5^ ± 0.6	7 × 10^3^ ± 0.5	–
FG + TA + BA	7.8 × 10^7^ ± 0.7	5.2 × 10^5^ ± 0.4	4.5 × 10^2^ ± 0.8	1.2 × 10^11^ ± 0.3

*Results of F. graminearum sporulations in response to the axenic and co-culture. Results are means of five replicates for each experiments; the value given is the standard error of the mean*.

### Induction of the Wheat Seed Germination by the Co-cultivation of *T. asperellum* and *B. amyloliquefaciens*

In the current study, it was observed that the seeds treated with the axenic and co-culture of *T. asperellum* and *B. amyloliquefaciens* were essentially higher than control (untreated seeds) (Table 5). Treatment TA + BA was found to be significantly superior and effective with 3.76 and 6.48 cm of the shoot and root length of wheat variety *Ningmai* 13 compared to the control, which was followed by TA (3.34 and 5.56 cm) and BA (2.08 and 4.22 cm). The positive impact of seed treatment was also documented for seedling length, wet weight, dry weight and vigor index. TA + BA treatment excelled overall significant superior performance by contributing seedling length (10.24 cm), wet and dry weight (2.66 and 0.65 mg) and vigor index (984).

**Table 5:** Effect of axenic and co-culture of *T. asperellum* and *B. amyloliquefaciens* on wheat seed germination.

Treatments	Shoot length (cm)	Root length (cm)	Seedling length (cm)	Seedling wet weight (mg)	Seedling dry weight (mg)	Vigor index
Control	1.8 ± 0.03^b^	4.04 ± 0.02^c^	5.84 ± 0.03^d^	1.76 ± 0.05^d^	0.59 ± 0.05^b^	584+0.03^d^
TA	3.34 ± 0.05^a^	5.56 ± 0.03^c^	8.9 ± 0.05^c^	2.46 ± 0.08^b^	0.65 ± 0.03^b^	890 ± 0.05^b^
BA	2.08 ± 0.06^b^	4.22 ± 0.01^b^	6.3 ± 0.06^a^	3.10 ± 0.09^c^	0.73 ± 0.08^a^	630 ± 0.06^c^
TA + BA	3.76 ± 0.06a	6.48 ± 0.01^a^	10.24 ± 0.06^b^	2.66 ± 0.05^a^	0.65 ± 0.07^b^	984 ± 0.06^a^

*Results are means of 10 replicates for each treatment; the value is the standard error of the mean. Different superscripts in the same column are significantly different (P < 0.05) based on the ANOVA.*

### Induction of the Wheat Growth and Bio-Control Activity by the Co-cultivation of *T. asperellum* and *B. amyloliquefaciens*

**FIGURE 7:**
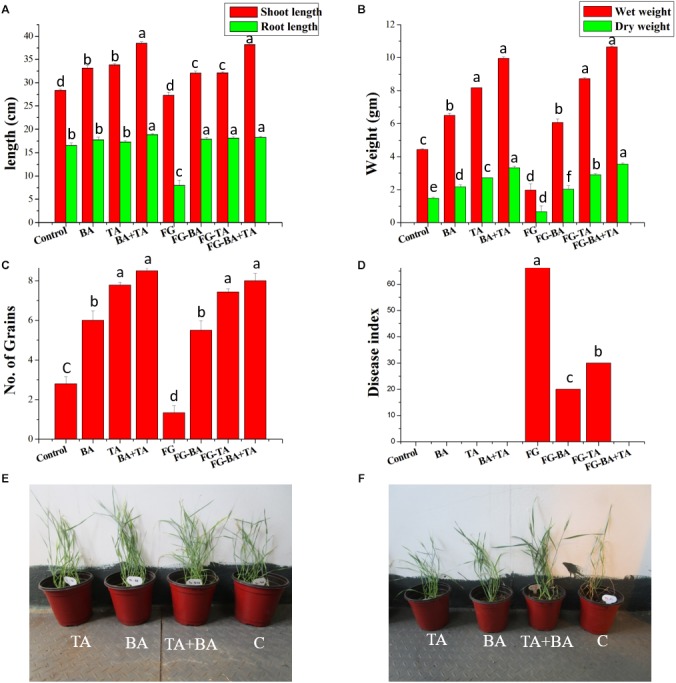
Effect of axenic and co-culture of *T. asperellum* and *B. amyloliquefaciens* on the plant growth and biological control of *F. graminearum* in green house conditions. **(A)** Shoot and root length of different treatments **(B)** wet and dry weight of wheat plants **(C)** number of grains per plant **(D)** disease index of *F. graminearum* in control and treated **(E)** Wheat plants grown in pots treated with TA (*T. asperellum*); BA (*B. amyloliquefaciens*) TA + BA (*T. asperellum* and *B. amyloliquefaciens*) C (control). **(F)** Wheat plants grown in pots containing *F. graminearum* treated with TA; BA; TA + BA; C (control). Results are means of five replicates for each treatment; the value in parentheses is the standard error of the mean. Different letters above the bars are significantly different (*P* < 0.05) based on the ANOVA.

The axenic and co-culture of *T. asperellum* and *B. amyloliquefaciens* were significantly increased the emergence of wheat seedlings compared with the control, which is also consistent with the increased emergence with the presence of *F. graminearum* (Figure 7A). Compared to the axenic culture of *T. asperellum* (TA) and *B. amyloliquefaciens* (BA), the co-culture of TA + BA resulted in the highest plant growth and emergence of wheat grains both in the presence and absence of *F. graminearum* (Figure 7B). similarly, the wet and dry weight of the plants treated with TA + BA was also significantly increased compared to the axenic culture (Figure 7B,C). 7 days after pathogen infection, all treatments were observed to ensure biocontrol efficiency compared to the control. The wheat plants treated with co-culture of *T. asperellum* and *B. amyloliquefaciens* showed healthy with fewer symptoms in root compared with the control treatment, which was only inoculated with the pathogen (Figure 7D–F). Followed by, the axenic culture of *T. asperellum* and *B. amyloliquefaciens* effectively controlled root rot, and the disease index (Figure 7D).

## Discussion

Co-cultivation methods have long been used to study the interactions between microbial populations. Recently, Co-cultivation has become a particular interest to study the changes in metabolic systems. Further, microbial co-cultivation is an approach motivated by inter-microbial competition and communication for the stimulation of novel metabolites by the activation of silent genes (Netzker et al., 2015). The usage of microbial consortium in the agriculture system may improve efficacy, stability and uniformity of the microbes in the plant disease control and plant growth promotion under diverse soil and environmental conditions (Stockwell et al., 2011). Similarly, co-culture system also enhances the plant growth and defense potential. The application of microbial co-culture stimulates the antioxidant enzyme activities and the phenylpropanoid pathway increases the total phenolics, proline, and pathogenesis-related (PR) proteins in the plants. Sarma et al. (2015) reviewed the consortium of biological control agents (BCAs) to control plant diseases in comparison with the use of individual agents. The beneficial effect of *T. asperellum* and *B. amyloliquefaciens* includes protection against pathogens, growth promotion, induction of disease resistance, and production of bioactive compounds with antibiotic and antitumor properties (Brunner et al., 2005; Xie et al., 2018). The previous study on the co-cultivation of *B. amyloliquefaciens* and *T. asperellum* inhibited the growth of *B. cinerea* (Wu et al., 2018). Hence in the present study, stimulation of diverse cellular processes such as sporulation, secondary metabolism, enzyme productions in the co-culture of *T. asperellum* GDFS1009 and *B. amyloliquefaciens* 1841 were studied to evaluate its effect on biocontrol, and plant growth.

Selection and optimization of the culture medium are expected to improve the growth of both *B. amyloliquefaciens* and *T. asperellum* for future biocontrol agent industry production. Initially, basic approaches like the screening of different media were used to enhance the growth of both cultures. Taking into consideration of three different mediums (NB, TY, and YMC), YMC enhanced the growth of both *B. amyloliquefaciens* and *T. asperellum*. The results inferred that yeast extract, molasses, and corn gluten meal enhance the growth of both *T. asperellum* GDFS1009 and *B. amyloliquefaciens* 1841. Yeast extract contains less complex mixes, for example, amino acids and peptides with the rich wellsprings of nutrients particularly those having a place with B-complex. Molasses contains huge measures of sugars, nutrients, and minerals, while corn gluten meal contains about 65% of rough proteins. It uncovers that the YMC medium containing all supplements, for example, carbohydrates, proteins, vitamins, and minerals. Thus, yeast extract, molasses, and corn gluten meal were chosen and further upgraded by utilizing Central Composite Design. The CCD results uncovered a high comparability between the anticipated and test results, which mirrored the exactness and appropriateness of RSM to upgrade the growth of *T. asperellum* GDFS1009 and *B. amyloliquefaciens* 1841 in submerged culture. The growth of the *B. amyloliquefaciens* and *T. asperellum* increased with the increment in each parameter up to the certain level, after which it was declined. This could be because of the loss of supplement in culture medium. The surface diagrams inferred the ideal estimations of yeast extract, molasses and corn gluten meal for the greatest growth of *T. asperellum* (8.2636 × 10^9^), and *B. amyloliquefaciens* (17.0795 × 10^11^) was 2 g each. So as to approve the sufficiency of the model, confirmation tests were directed under anticipated ideal maturation conditions. The outcomes have demonstrated that the model is acceptable.

Conidiation of the *Trichoderma* is influenced by light, mechanical injury and environmental conditions such as nutrients and pH. The photoreceptor complex BLR-1/BLR-2, ENVOY, VELVET, and NADPH oxidases have been involved in the conidiation process. In performance with these genes, conserved signaling pathways, such as heterotrimeric G proteins, MAPKs and cAMP-dependent protein kinase A (cAMP-PKA) are also involved in the conidiation process (Carreras-Villasenor et al., 2012). Hence, in the present investigation, the expression of blue-light-regulated genes (BLR1 and BLR2), ENVOY (ENV 1), velvet (Vel1) Mitogen-Activated Protein Kinase (*TMKa*) and G protein receptor 1 (GPR1) in the axenic and co-culture of the *T. asperellum* in YMC medium was studied. All these genes were upregulated in the co-culture. These gene expressions were correlated with the increase in sporulation of co-culture. Hence, the technology on the co-cultivation of the *T. asperellum* and *B. amyloliquefaciens* could be used to increase the *Trichoderma* spore concentration. These findings correlated well with the model predicted by the Carreras-Villasenor et al. (2012). In the present study, the nutrient-rich medium and the co-cultivation strategy influenced the expression of blue-light-regulated genes (BLR1 and BLR2), ENVOY (ENV 1), velvet (Vel1), Mitogen-Activated Protein Kinase (*TMKa*) and G protein receptor 1 (GPR1). But the mechanism behind the activation of these genes is unknown.

The assessment of signaling pathways of *Trichoderma* revealed that heterotrimeric G-proteins and mitogen-activated protein (MAP) kinases influence biocontrol-relevant mode of actions such as the synthesis of hydrolytic enzymes and antibiotics (Zeilinger et al., 2016). MAPK signaling was also found to be implicated in stimulation of plant systemic resistance in *Trichoderma virens* (Mukherjee et al., 2003) and stress response in *Trichoderma harzianum* (Delgado-Jarana et al., 2006). The role of cAMP pathway during *Trichoderma* biocontrol exposed stimulation of mycoparasitism-associated coiling and chitinase and secondary metabolites (Omann and Zeilinger, 2010). The present study found that expression of Mitogen-Activated Protein Kinase (*TMKa*) and G protein receptor 1 (GPR1) in co-culture was induced by the *B. amyloliquefaciens*. The over expression of Mitogen-Activated Protein Kinase (*TMKa*) and G protein receptor 1 (GPR1) might be responsible to increase the antagonism and biocontrol activity of the *T. asperellum* and *B. amyloliquefaciens* co-culture.

The expression of genes concerned in the production of secondary metabolites is directed by a hierarchical network of regulators that respond to multiple environmental signals (Netzker et al., 2015). To address this question, we examined changes in secondary metabolite gene expression of the *T. asperellum* in respond to the *B. amyloliquefaciens*. We found that secondary metabolite gene such as non-ribosomal peptide synthetase (NP1 and NP2), Putative ferrichrome synthetase (NP3), Cytochrome P450 (Tri 13) 1, OMT and Polyketide synthetase (PK1 and PK2) were expressed at higher levels in co-culture. Chettri et al. (2012) and Lind et al. (2015) explained that the expression of velvet protein complex alters expression of secondary metabolite gene clusters and developmental processes in many fungi. Similarly, in the present investigation both velvet and secondary metabolite related genes were up-regulated in the co-culture. Further, the expression of velvet genes showed the relationship with sporulation and the regulation of NRPS gene expression. It supports the assumption that fungal secondary metabolism is synchronized by a network of transcriptional regulators with response to different environmental stimuli.

The present study affords an involvement in understanding the role of a *T. asperellum* protein related to ROS production in the interaction between *B. amyloliquefaciens.* It has been established that *NOX* genes are stimulated by the existence of pathogens in mammals and plants (Wang et al., 2016). Montero-Barrientos et al. (2011) detected the motifs for binding the regulators of oxygen metabolism, sexual development, and PR proteins in the *nox1* promoter region and suggest that *NOX* proteins as an important enzyme for sexual development. It was clear that the polysaccharides present in the cell wall of fungal pathogens and plant materials would induce the expression of NOX gene. In the present study, we observed that the NOX gene was also induced by the bacteria. ROS liberation by NADPH oxidases is a common signaling system among all organisms. The sequence of ROS scavenging systems such as ascorbate peroxidases, glutathione, superoxide dismutases, and catalases, associated with NADPH oxidases to maintain the ROS homeostasis in cells (Montero-Barrientos et al., 2011). Similarly, in the present study, we observed the sequential up-regulation of catalase gene in response to the expression of *NOX* gene in the co-culture. The transcriptomic response of *T. harzianum* T34 *NOX1* over expression with *Pythium ultimum* upregulated the genes encoding proteases, including aspartic, subtilisin serine, and trypsin-like proteases (Montero-Barrientos et al., 2011). Similarly, we observed that induction of genes encoding aspartyl protease, trypsin-like protease, chitinase, β-1,3-glucanase, β1,6-glucanase, β-1,4-glucanase and *N*-acetyl-glucosaminidases in co-culture.

The optimistic effect of co-cultivation of fungi on the liberation of enzymes has been studied previously (Lima et al., 2016). However, no reports have illustrated the effects of co-cultivating the two most agriculturally important bacteria and fungus (*T. asperellum* and *B. amyloliquefaciens*) on the enzyme production. In this paper, we have addressed this question. Several co-cultivation technologies resulted in enhanced activity for numerous enzymes, but often not for all enzymes. This implies that the co-cultivation does not enhance the protein secretion but rather it enhances the specific enzymes, which was concluded by the estimation of total extracellular protein in co-culture medium. This was also dependent on the carbon source supplemented in the medium. Hence, we used a nutrient-rich media (YMC). The result revealed that co-cultivation can result in specific up-regulation of genes encoding the enzymes such as *N*-acetyl-glucosaminidases (NAG1 and NAG 2), β-1,3-glucanase (*BGN* 13), β-1,4-glucanase (EG1), β1,6-glucanase (*BGN* 16), NAG 1, chitinase (ECH 42) and aspartyl protease (*Pap* A) and enzyme production such as chitinase, β 1–3 glucanase, cellulase and protease compared to the axenic incubations. Similarly, Copete-Pertuz et al. (2019) identified a triple combination inoculated with *Trichoderma viride* and *Aspergillus terreus* into a 7th day preculture of *Leptosphaerulina* sp. improved ligninolytic enzymes production when compared to the *Leptosphaerulina* sp. monoculture.

Hydrolytic enzymes and antibiotics of *Trichoderma* are the most essential components to kill other fungi (Hermosa et al., 2013). In the present study, the highest antagonistic activity and the biocontrol efficiency of the co-culture observed might be due to the overexpression of hydrolytic enzymes and secondary metabolites in the co-culture conditions. In addition to hydrolytic enzymes and antibiotics, proteases like Prb1/Sp1 in co-culture and axenic culture of *T. asperellum* was induced during the stimulated antagonism assay and proved definitive roles in biocontrol.

*Bacillus* strains are generally studied for their valuable role in plant growth and biological control of plant disease and pest (Mota et al., 2017). In the present study, the antagonism, biocontrol and plant growth were induced in the axenic culture of *B. amyloliquefaciens* compared to the control. *B. amyloliquefaciens* produce polyketides such as macrolactin, difficidin, and oxidifficidin (Goodson et al., 2017). These compounds have been used as biocontrol agents. Hence, we accessed the gene expression of *Loa P* [intrinsic terminators located within the polyketide synthase (PKS) gene cluster encoding for the antibiotic difficidin (*dfn*)], dfnA, dfnG, dfnM (beginning, middle and end of the difficidin operon) MLN a, MLN d, MLN i (beginning, middle and end of the macrolactin operon). The results inferred that The *B. amyloliquefaciens* secondary metabolite genes were not expressed in the co-culture conditions. The reason behind the down-regulation of these genes was unknown.

This study further revealed the highest potential of *Bacillus* and *Trichoderma* co-cultivations on the plant growth promotion. The enzyme 1-aminocyclopropane-1-carboxylate (ACC) deaminase (ACCD, EC 4.1.99.4) is widespread among rhizobacteria (Glick, 2014) and reduce ACC, the precursor of ethylene, to α-ketobutyrate and ammonia. Thereby, it induces the growth of plant and root under biotic and abiotic stress (Glick, 2014). For the first time, Zhang et al. (2016) analyzed the ACC deaminase gene of the biocontrol fungus *T. asperellum* ACCC30536. In this present investigation, the expression of ACC deaminase was up-regulated in co-cultivation. This was supported by the seed germination assay and plant growth.

Comprehensive interactions take place between filamentous fungi and other living materials mostly with the help of volatile metabolites. Secondary metabolites of fungi could play a role in plant defense. This proposes that the volatile metabolite contributes to the antifungal activity (Leylaie and Zafari, 2018). Hence, in the present investigation, the changes in the secondary metabolites in axenic and co-culture were detected using the GC-MS analysis. The most abundant metabolite originally characterized by Collins and Halim (1972) was identified to be 6-pentyl-alpha-pyronein (6-PP). It was found in both axenic and co-culture of *T. asperellum*. It was identified as one of the key metabolites of *T. asperellum*. This compound is oxygen-containing heterocyclic compound which produces coconut odor, a typical characteristic of the *Trichoderma* (Fadel et al., 2015). This compound induces the cellular function, antibiosis, plant growth promotion and defense response (Fadel et al., 2015). α-cuprenene is a basic structure required for antimicrobial sesquiterpene quinone (Melillo et al., 2013) was found only in the co-culture medium. This might be responsible for the production of more terpenes in the co-culture medium.

Iturins are a group of antifungal, cyclic lipopeptides, produced by *Bacillus* (Meena and Kanwar, 2015). The iturin group of compound includes iturin A–E, bacillomycin D, F, and L, and mycosubtilin. These groups of compound contain seven α-amino acids (A_1_–A_7_) and one distinctive β-amino fatty acid (βAA). In the present investigation the alpha amino acids and amino fatty acids were detected in the *B. amyloliquefaciens* axenic culture and co-culture indicated the production of iturin like compounds. Kai et al. (2008) observed that dual culture system of bacteria with fungi and *Arabidopsis thaliana* emits volatiles and act as antibiotics. Similarly, in the present investigation, the co-culture system produced the new volatile compounds such as alkanes, alkenes, alcohols, esters, ketones, sulfur-containing compounds and terpenoids.

The heterocyclic compound, 1,2-Benzisothiazol-3(2H)-one was detected only in the co-culture. 1,2-Benzisothiazol-3(2*H*)-one play a major role in several pharmaceutical applications with broad spectrum bioactivity (Mahmoud et al., 2011; Lai et al., 2013). Amongst, appropriately substituted 1,2-benzisothiazol-3(2*H*)-ones have been appeared to show potent antifungal and antivirus activity (Dou et al., 2011; Tiew et al., 2012). The production of 1,2-Benzisothiazol-3(2H)-one in co-culture medium could be also involved in the improvement of antifungal and biocontrol activity of *T. asperellum* in respond to the *B. amyloliquefaciens* co-culture.

Proline plays a vital role in plant growth and development. It is essential for several cell wall proteins that play significant roles in plant growth such as cell wall signal transduction cascades, plant development and stress tolerance (Kavi Kishor et al., 2015). In the present investigation, the L-Proline, *N*-pivaloyl- ethyl ester was found in only co-culture system and this might be responsible for the highest seed germination, biocontrol and plant growth (Table 5 and Figure 7). Similarly, Wu et al. (2018) observed that the proline metabolism was induced in the co-culture of *B. amyloliquefaciens* ACCC11060 and *T. asperellum* using co-inoculation method.

The global biopesticide and biofertilizer market is constantly rising due to guidelines of agricultural legislations and regulations (Woo et al., 2014). The current research trend toward the development of biopesticide and biofertilizer focused toward the formulation of microbial consortia by mixing two separately grown microbial culture. Therefore, For the first time, in the present study the two different plant growth promoting and biological control agents were synergistically co-cultivated and optimized using the economic medium to reduce the production cost of two beneficial microbes at same time. Despite the different nature of two agriculturally important microbes, the co-cultivation of *B. amyloliquefaciens* 1841 and *T. asperellum* GDFS1009, stimulated different signaling pathways and enhanced the production of antagonistic and mycoparasitism related enzymes, secondary metabolites, and plant growth promoting compounds. Further, the greenhouse study confirmed that the biocontrol and plant growth was more effective in the plants treated with co-culture. The enhanced production of the biologically active compounds and plant growth recommended that this co-cultivation technology could be used as the next generation biopesticide and biofertilizer. Further, the scale-up of this co-cultivation technology in the fermentor and field study will facilitate the use in agriculture sector.

## Author Contributions

VK, and JC conceived and designed the experiments and wrote the Manuscript. VK performed the experiments. VK, JS, TL, MV, and JC analyzed the data.

## Conflict of Interest Statement

The authors declare that the research was conducted in the absence of any commercial or financial relationships that could be construed as a potential conflict of interest.
