# Supplementary material for: Co-cultivation of Trichoderma asperellum GDFS1009 and Bacillus amyloliquefaciens 1841 Causes Differential Gene Expression and Improvement in the Wheat Growth and Biocontrol Activity
Source: Front Microbiol. 2019 May 16;10:1068. doi: 10.3389/fmicb.2019.01068 (PMC6532653; doi:10.3389/fmicb.2019.01068)
Supplement: Supplementary file 1 [file Data_Sheet_1.docx]

Supplementary Material

**Supplementary Table 1. Sequences of the primers used for the real-time PCR.**

| Gene | Forward (5’-3’) | Reverse (5’-3’) | Reference |
| --- | --- | --- | --- |
| *VEL* 1 | CGAGGAGGGCAAGGACATTAC | GCAGGAACACCAGTCAGGATG | (Bazafkan et al., 2017) |
| *TMK* | CCAGCCCGACCATCATGTCTC | CGCATAATCTCTTGGTAAATCAGTTG | (Mukherjee et al., 2003) |
| *GPR* 1 | TTGATCCAGACCTTCATGCCAGC | CATAAAAGGCCGCGACACGAA | (Brunner et al., 2008) |
| *BLR* 1 | GGGATGACAGCCGAAC | TCAGCTCCCGCGTGAC | (Casas-Flores et al., 2004) |
| *BLR* 2 | CATTGCGGCTGCTAGG | GTCCCTTTCGCCATTC |  |
| *ENV1* | GCCCTCTCGACTGCTCCGTC | CGACCCATGATCTCGGGGGC | (Bazafkan et al., 2017) |
| *NAG* 1 | TCATTGGCGGTGAACTCG | TCGTCCCGACCACCAGAT | (Yang, 2017) |
| *NAG* 2 | CAAGAGGACCATTGACGC | GCTTAGGCAGTGAAGGGAT |  |
| *BGN13* | GCAGCCCTCAACAAGGTG | AGAGCCAACGGGAACCAC |  |
| *BGN* 16 | ATGAGTCTCCTGGAATGG | GTTGGGATGTAGTTGAGGT |  |
| *EG1* | CTACTTCGGCCCCGGAGA | GGTGATGCTGACGAGGTTG |  |
| *ECH* | AGTACCCTGCCGATGACA | TAGCCTGGAGCGTATTGC |  |
| *PAP* A | TGGACAGCATCAACACCG | ACTGAGCAGATGAGACCTGGCCGTAGT | (Chet et al., 2004) |
| *PAP* B | GACAGGACATTGCCTGGACT | GACTGGAC TCCTCCGTACCA |  |
| *AF* | ATGGAGCTTAAAGCACTCAG | TCAGCGCTGGAGAGTTAGC |  |
| *NOX* | CACCACCTGTTCATCCC | GTCAAATGGCGAGAATCC | (Montero-Barrientos et al., 2011) |
| *CAT* | ACTGCATTGTCCGTTTCT | AGTTGCCCTCCTCTGTG |  |
| *ACC* | GACCTGCTCCACCATCTTCC | CAGTGGAGTTGCCGACAAAG | (Viterbo et al., 2010) |
| *NP1* | GCGAATCAGAACAACAGCC | CATAGCCGTTCAGCCCATC | (Mukherjee and Kenerley, 2010) |
| *NP2* | CGTCCGTGGATATCCAGGC | GCCATCCGTATAGCCTGAC |  |
| *NP3* | CAAGACGCGTTTCACCTTCTTG | CGCTGTCCATTTGATCTCGC |  |
| *Tri* 13 | CATGGATGCAATCTGGGCCATTGT | TGGCCGCCCATAATAAATCCGAGA |  |
| *OMT* | CACTGTTGCACAGGCTGTTCCATT | AAGTTGTACCACTGCTCCTCGGTT |  |
| *PK1* | AAGACAATCCAACCTATCGGGCCA | TCTGCAACATCACAAGGCACAACG |  |
| *PK2* | CGCGCAACTTCAACGCTCTTACAA | TCATAGGCACAAATACCTCCCGCA |  |
| *18S rRNA* | GGTGGAGTGATTTGTCTG | CTTACTAGGGATTCCTCG | (Tisch et al., 2011) |
| *LoaP* | GCAGGCTCTCTACTCAATCATAC | CAGCACATATCCCGGAAACA | (Goodson et al., 2017) |
| *DfnA* | CCCATTACCTATGCCGAAAGA | TCTTCGTTCCCTAATCTCATTCC |  |
| *DfnG* | ACAGCGAACTGACGGAATAC | GGCTGAGCTCAAGCTGATAA |  |
| *DfnM* | GGGCTGTCAGAAGAGTTTGT | ATTTCCGTAACCCGTCAGAAG |  |
| *DlnA* | TGATGCTGTTGCAGGACATAG | CTCGGAAGCCTCACTCATTAAC |  |
| *DlnD* | TTGGAAACGGAAGAAGGGATAG | ACGAACCGGAAGCGTATTT |  |
| *DlnI* | GAAGGCACCTTCTCACATCTT | ACGGCATGAGGATGAAACTC |  |
| *16S rRNA* | TCGCGGTTTCGCTGCCCTTT | AAGTCCCGCAACGAGCGCAA | (Cui et al., 2018) |

**Supplementary Table 2. The metabolites identified from the axenic and co- culture of *T.asperellum* and *B. amyloliquefaciens* Using GC-MS.**

| **Retention Time** | **Compound** | ***TA*** | ***BA*** | **TA+BA** | **Classification** |
| --- | --- | --- | --- | --- | --- |
| 11.895 | Cyclobutane, methylene - |  |  | + | Nitrogen containing compounds |
| 16.496 | Undecanoic acid, 11-amino- |  |  | + | Amino fatty acids |
| 17.528 | 4-Spirohexanone, 5,5-dichloro- |  |  | + | Hetrocyclic compounds |
| 18.242 | 2-Butenethioic acid, S-[2-(acetylamino)ethyl] ester |  |  | + | Sulphur containing compounds |
| 19.273 | Ethylene oxide |  |  | + | Ethers |
| 19.590 | 1,5-Pentanediol |  |  | + | Alcohols |
| 20.780 | Propane |  |  | + | Alkanes |
| 21.256 | 1-Hexanamine |  |  | + | Amines |
| 22.446 | Hexanenitrile, 6-amino- |  |  | + | Cyclic peptide |
| 23.002 | Dimetridazole |  |  | + | Hetrocyclic compounds |
| 25.644 | 2-Coumaranone | _+_ | + | + | Hetrocyclic compounds |
| 26.437 | 4-Methoxycarbonyl-4- butanolide | + |  |  | Hetrocyclic compounds |
| 27.127 | 1,3-Dioxolane, 2-ethyl-2,4,5-trimethyl- |  | + | + | Hetrocyclic compounds |
| 27.921 | Ethylene oxide |  |  | + | Ethers |
| 28.341 | dl-Mevalonic acid lactone |  | + |  | Hetrocyclic compounds |
| 28.555 | Benzenepropanamide, N-(4-aminobutyl)-3,4-dihydroxy- |  |  | + | amines |
| 29.134 | 4(1H)-Pyridone |  | + |  | Hetrocyclic compounds |
| 29.349 | 4H-[1,2,4]Triazolo[1,5-a]pyrimidin-7-one, 5-methyl- |  |  | + | Hetrocyclic compounds |
| 29.904 | 1-Piperazinecarboxylic acid, 4-(2-furanylcarbonyl)-, ethyl ester |  |  | + | Nitrosamines |
| 30.063 | 3-Methyl-3,5--(cyanoethyl)tetrahydro-4-thiopyranone |  |  | + | Hetrocyclic compounds |
| 30.166 | Cyclohexasiloxane, dodecamethyl- |  | + |  | Polymers |
| 30.483 | Phenol, 2-methyl- | + |  | + | Aromatic hydrocarbon |
| 31.038 | Phenol, 2,6-dimethoxy- |  | + |  | Aromatic hydrocarbons |
| 31.435 | 2H-Pyran-2-one, 3-acetyl-4-hydroxy-6-methyl- |  | + |  | Hetrocyclic compounds |
| 31.594 | 6-Pentyl-2H-Pyran-2-One | + |  | + | Pyrone derivative |
| 31.911 | Benzenepropanoic acid, .alpha.-hydroxy-, methyl ester | + | + | + | Aromatic hydrocarbon |
| 32.149 | 8-Dodecenoic acid, 11-hydroxy-, methyl ester, [s-(E)]- |  | + |  | Fatty acid esters |
| 32.387 | 3-Hydroxy-1,7-dioxaspiro[5.5]undecane | + |  | _+_ | Polyketides |
| 32.863 | Phenelzine |  |  | + | Amine |
| 33.316 | Silane, 9-anthracenyltrimethyl- |  |  | + | Halogenated hydrocarbons |
| 33.815 | 5,7-Dimethylenebicyclo[2.2.2]oct-2-ene |  |  | + | Fatty acid ester |
| 34.847 | Piperidine, 1-methyl- | + |  | + | Hetrocyclic compounds |
| 35.402 | trans-7-Carboxy-bicyclo[4.3.0]non-3-ene | + |  |  | Hetrocyclic compounds |
| 35.378 | (1R,4R,4aS,8aR)-4,7-Dimethyl-1-(prop-1-en-2-yl)-1,2,3,4,4a,5,6,8a-octahydronaphthalene |  |  | + | Terpenes |
| 36.251 | alpha.-Cuprenene |  |  | + | Hetrocyclic compounds |
| 36.354 | Phthalic acid, hexyl 2,2,2-trifluoro-1-phenylethyl ester |  | + |  | Hetrocyclic compounds |
| 36.751 | Cyclohexene, 3-bromo- | + |  |  | Alkane |
| 36.886 | 1H-3a,7-Methanoazulene, 2,3,4,7,8,8a-hexahydro-3,6,8,8-tetramethyl-,[3R-(3. alpha., 3a. beta., 7.beta., 8a. alpha.)]- |  |  | + | Terpenes |
| 37.703 | Phenol, 2,4-bis(1,1-dimethylethyl)- | + | + | + | Aromatic hydrocarbons |
| 38.258 | Acetamide, N-(2-phenylethyl)- | + |  | + | Amines |
| 38.575 | D-Alloisoleucine, N-acetyl- |  | + |  | Amino acid |
| 39.028 | (3R,6S)-3-Hydroxy-1,7-dioxaspiro[5.5]undecane |  |  | 1. + | 1. Polyketides |
| 39.765 | beta.-Bisabolene |  | + | + | Terpenes |
| 40.162 | 1H-Cycloprop[e]azulene, 1a,2,3,5,6,7,7a,7b-octahydro-1,1,4,7-tetramethyl-, [1aR-(1a.alpha.,7.alpha.,7a.beta.,7b. alpha.)]- | + |  |  | Hetrocyclic compounds |
| 40.638 | 5-Octadecene, (E)- | + |  |  | Alkenes |
| 40.955 | N-Formylmorpholine |  | _+_ |  | hetrocyclic compounds |
| 40.876 | Benzene, 1,2,4,5-tetramethyl- |  |  | + | hetrocyclic compounds |
| 41.273 | 3-Hydroxy-4-methoxybenzoic acid | + |  |  | Ethers |
| 41.590 | 2-Propen-1-ol, 3-phenyl- |  |  | + | Alcohols |
| 41.408 | 1,3,5,7-Cyclooctatetraene |  |  | + | Aromatic hydrocarbon |
| 41.884 | Phenol, 3,4,5-trimethoxy- |  |  | + | Ether |
| 41.908 | Benzeneacetic acid, 4-hydroxy | _+_ | + |  | Hetrocyclic compounds |
| 42.519 | 3-((1S,5S,6R)-2,6-Dimethylbicyclo[3.1.1]hept-2-en-6-yl)propanal |  |  | + | Aldehyde |
| 42.836 | Benzofuran, 2,3-dihydro-2-methyl- |  |  | + | Peptide |
| 43.098 | 2(3H)-Furanone, dihydro-5-(2-octenyl)-, (Z)- | + |  | + | Fatty acids |
| 43.256 | L-Histidine, 1-methyl- |  | + |  | Amino acid |
| 43.574 | Pyrrolidine, 3,3-dimethyl-4-[(2-methoxycarbonyl)ethyl]-2,5-dione- | + |  | + | Hetrocyclic compounds |
| 43.709 | Benzene, 1-(1,5-dimethyl-4-hexenyl)-4-methyl |  |  | + | Terpenes |
| 43.970 | Cyclohexanone, 3-(4-hydroxybutyl)-2-methyl- |  | + |  | Hetrocyclic compounds |
| 44.288 | Methyl tetradecanoate |  | + |  | Fatty acid |
| 44.526 | 9-Decenoic acid | + |  |  | Fatty acid |
| 44.684 | N,N'-Di-p-tolyl-formamidine |  | + |  | Sulfenamides |
| 44.843 | beta.-curcumene | + |  |  | Aliphatic compound |
| 45.160 | L-Phenylalanine, N-acetyl-, methyl ester |  | + |  | Aminoacid |
| 45.636 | Methyl tetradecanoate |  |  | + | Fatty acid |
| 45.716 | 1,4-Benzenediol, 2,5-bis(1,1-dimethylethyl)- | _+_ |  | + | Aromatic hydrocarbon |
| 46.351 | Naphthalene, 1,1'-(1,2-ethanediyl)bis[decahydro- | + |  |  | Aromatic hydrocarbons |
| 46.327 | (6R,7R)-Bisabolone |  | + | + | Terpenes |
| 47.065 | Anthracene | _+_ |  |  | Aromatic hydrocarbons |
| 47.279 | Benzene, 1-(1,5-dimethyl-4-hexenyl)-4-methyl- |  |  | + | Terpenes |
| 47.937 | 5-Octadecene, (E)- | + |  | + | Alkenes |
| 48.37 | Methyl 9-methyltetradecanoate |  | + |  | Fatty acid |
| 48.71 | 2,3-Diaminophenol |  | + |  | Halogen |
| 48.810 | Benzonitrile, 4-[(2-hydroxyphenylimino)methyl]-benzylidenamino)- | + |  |  | Hetrocyclic compounds |
| 50.214 | L-Proline, N-pivaloyl-, ethyl ester |  |  | + | Amino acid |
| 50.397 | Pentadecanoic acid | + | + | + | Fatty acid |
| 49.841 | Phenol, 3,5-dimethoxy- | + |  | + | Aromatic hydrocarbon |
| 51.190 | Hexadecanoic acid, methyl ester |  | _+_ |  | Fatty acid ester |
| 51.960 | Cyclohexanone, 5-methyl-2-(1-methyl-1-phenylethyl) |  |  | + | Hetrocyclic compounds |
| 51.984 | 1,3-Dithiane, 2-octyl- | + |  |  | Halogens |
| 52.277 | 2,5-Cyclohexadien-1-one, 2,6-bis(1,1-dimethylethyl)-4-ethylidene- |  |  | _+_ | Ketones |
| 52.595 | Pentadecanoic acid, 14-methyl-, methyl ester |  |  | + | Compounds containing nitrogen |
| 52.301 | 7,9-Di-tert-butyl-1-oxaspiro(4,5)deca-6,9-diene-2,8-dione | + |  |  | Lactone |
| 52.697 | Methyl palmitate |  | + |  | Fatty acid |
| 53.150 | Benzaldehyde, 4-methoxy-3-(4-methylphenylthiomethyl)- |  |  | + | Hetrocyclic compounds |
| 53.174 | L-Proline, N-valeryl-, octyl ester |  |  | + | Hetrocyclic compounds |
| 54.602 | n-Hexadecanoic acid | + | + | + | Fatty acid |
| 55.316 | 3,5-Dimethoxytoluene | + |  |  | Ethers |
| 55.950 | Heptadecanoic acid, methyl ester | + |  |  | Fatty esters |
| 56.585 | Naphthalene, 6-decyl-1,2,3,4-tetrahydro- | + |  |  | Aromatic hydrocarbons |
| 56.323 | Tetradecanoic acid |  |  | + | Fatty acid |
| 57.14 | Heptadecanoic acid | + | + | + | Fatty acid |
| 57.537 | 1-Octadecene |  |  | + | Alkenes |
| 57.775 | 9-Octadecenoic acid, methyl ester, (E)- | + | + | + | Fatty acid ester |
| 58.64 | Methyl stearate | + | + | + | Fatty acid ester |
| 59.838 | 6-Octadecenoic acid |  | + | + | Fatty acid |
| 59.20 | Oleic Acid | + |  |  | Fatty acid |
| 59.83 | Octadecanoic acid | + |  | + | Fatty acid |
| 61.34 | 1,3-Cyclohexadiene-1-carboxaldehyde, 2,6,6-trimethyl- | + |  | + | Acyclic hydrocarbons |
| 62.13 | 2,5-Bis(1,1-dimethylbutyl)-4-methoxyphenol | + |  |  | Aromatic hydrocarbon |
| 62.93 | Eicosanoic acid, methyl ester | + |  |  | Fatty acid ester |
| 63.15 | Carbonic acid, octadecyl 2,2,2-trichloroethyl ester |  |  | + | Ester |
| 63.42 | cis-4-Hydroxy-3-methyldecanoic acid lactone |  |  | + | Hetrocyclic compound |
| 63.64 | n-Propyl 9,12-octadecadienoate |  | + |  | Fatty acid ester |
| 63.72 | Eicosanoic acid | + |  | + | Fatty acid |
| 64.28 | Cyclotetracosane | + |  |  | Carbo cyclic compounds |
| 64.33 | 1,2-Benzisothiazol-3(2H)-one |  |  | + | Hetrocyclic compounds |
| 65.20 | oxazepam, 2TMS derivative |  |  | + | Hetrocyclic compounds |
| 65.60 | 5-Isoxazolepropanamide, 3-chloro-N-(2,3-dihydro-1,4-benzodioxin-6-yl)- |  |  | + | Hetrocyclic compounds |
| 66.82 | 2(1H)-Phenanthrenone, 3,4,4a,9,10,10a-hexahydro-7-hydroxy-1,1,4a-trimethyl-8-(1-methylethyl)-, (4aS-trans)- | _+_ |  |  | Terpenes |
| 66.87 | 1-(3,3-Dimethylbutyn-1-yl)-2,2-dimethylcyclopropene |  |  | + | Organophosphorus |

**Supplementary figure 1. Inhibitory activities of axenic and coculture of *T. asperellum* and *B. amyloliquefaciens* fermentation liquor against plant pathogenic fungi. Where (TA) culture filtrate of *T. asperellum*; (BA) culture filtrate of *B. amyloliquefaciens*, and (TA+BA) culture filtrate of *T. asperellum* and *B. amyloliquefaciens***.


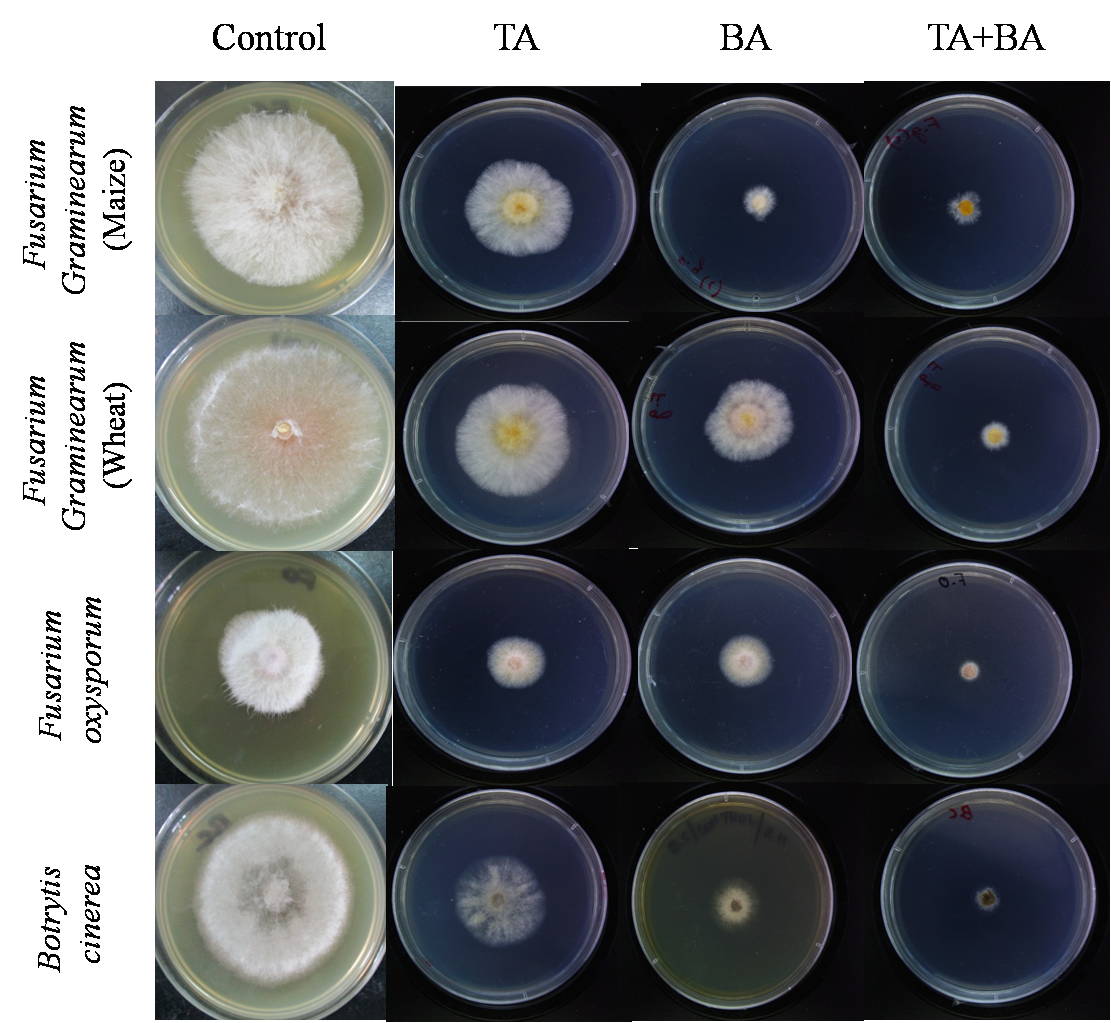


References

Bazafkan, H., Dattenböck, C., Stappler, E., Beier, S., and Schmoll, M. (2017). Interrelationships of VEL1 and ENV1 in light response and development in Trichoderma reesei. *PLOS ONE* 12**,** e0175946.

Brunner, K., Omann, M., Pucher, M.E., Delic, M., Lehner, S.M., Domnanich, P., Kratochwill, K., Druzhinina, I., Denk, D., and Zeilinger, S. (2008). Trichoderma G protein-coupled receptors: functional characterisation of a cAMP receptor-like protein from Trichoderma atroviride. *Current genetics* 54**,** 283-299.

Casas-Flores, S., Rios-Momberg, M., Bibbins, M., Ponce-Noyola, P., and Herrera-Estrella, A. (2004). BLR-1 and BLR-2, key regulatory elements of photoconidiation and mycelial growth in Trichoderma atroviride. *Microbiology* 150**,** 3561-3569.

Chet, I., Harel, M., and Viterbo, A. (2004). Isolation of two aspartyl proteases from Trichoderma asperellum expressed during colonization of cucumber roots☆. *FEMS Microbiology Letters* 238**,** 151-158.

Cui, W., Suo, F., Cheng, J., Han, L., Hao, W., Guo, J., and Zhou, Z. (2018). Stepwise modifications of genetic parts reinforce the secretory production of nattokinase in Bacillus subtilis. *Microbial biotechnology* 11**,** 930-942.

Goodson, J.R., Klupt, S., Zhang, C., Straight, P., and Winkler, W.C. (2017). LoaP is a broadly conserved antiterminator protein that regulates antibiotic gene clusters in Bacillus amyloliquefaciens. *Nat Microbiol* 2**,** 17003.

Montero-Barrientos, M., Hermosa, R., Cardoza, R.E., Gutiérrez, S., and Monte, E. (2011). Functional analysis of the Trichoderma harzianum nox1 gene, encoding an NADPH oxidase, relates production of reactive oxygen species to specific biocontrol activity against Pythium ultimum. *Applied and environmental microbiology* 77**,** 3009-3016.

Mukherjee, P.K., and Kenerley, C.M. (2010). Regulation of Morphogenesis and Biocontrol Properties in Trichoderma virens by a VELVET Protein, Vel1. *Applied and Environmental Microbiology* 76**,** 2345-2352.

Mukherjee, P.K., Latha, J., Hadar, R., and Horwitz, B.A. (2003). TmkA, a mitogen-activated protein kinase of Trichoderma virens, is involved in biocontrol properties and repression of conidiation in the dark. *Eukaryotic cell* 2**,** 446-455.

Tisch, D., Kubicek, C.P., and Schmoll, M. (2011). New insights into the mechanism of light modulated signaling by heterotrimeric G-proteins: ENVOY acts on gna1 and gna3 and adjusts cAMP levels in Trichoderma reesei (Hypocrea jecorina). *Fungal Genetics and Biology* 48**,** 631-640.

Viterbo, A., Landau, U., Kim, S., Chernin, L., and Chet, I. (2010). Characterization of ACC deaminase from the biocontrol and plant growth-promoting agent Trichoderma asperellum T203. *FEMS Microbiology Letters* 305**,** 42-48.

Yang, P. (2017). The gene task1 is involved in morphological development, mycoparasitism and antibiosis of Trichoderma asperellum. *Biocontrol Science and Technology* 27**,** 620-635.
